# Continuity in primary care: a critical but neglected component for achieving high-quality universal health coverage

**DOI:** 10.1136/bmjgh-2019-001435

**Published:** 2019-05-23

**Authors:** Dan Schwarz, Lisa R Hirschhorn, June-Ho Kim, Hannah L Ratcliffe, Asaf Bitton

**Affiliations:** 1 Primary Health Care, Ariadne Labs, Boston, Massachusetts, USA; 2 Division of Global Health Equity, Brigham and Women's Hospital Department of Medicine, Boston, Massachusetts, USA; 3 Northwestern University Feinberg School of Medicine, Chicago, Illinois, USA; 4 Division of General Internal Medicine, Brigham and Women's Hospital Department of Medicine, Boston, Massachusetts, USA

**Keywords:** continuity, primary care, primary health care, low- and middle-income countries, health systems, universal health coverage, quality, equity

**Summary box**

- Continuity is a critical but often neglected function of high-quality primary care and has three core domains: relational, informational and managerial continuity.
- Improving continuity is feasible in low-income and middle-income country health systems by using comprehensive empanelment systems or community-based follow-up programmes to improve retention in care.
- Continuity must receive more attention, measurement and improvement efforts, in order to achieve equitable, high-quality health for all.

## Introduction

Despite increasing attention to *what* the goals for universal health coverage are,^1^ the global health community still lacks clarity on *how* low-income and middle-income countries (LMICs) can strengthen health systems to reach these ambitious goals, while ensuring quality at the same time.^2^ In October 2018, the world commemorated the 40th anniversary of the Alma-Ata declaration and issued the Astana Declaration on Primary Health Care, clearly centralising the universal health coverage agenda within an overall framing of strong primary healthcare, and offering guidance for the way forward.^3^

To meet the Sustainable Development Goals^4^ and deliver quality universal health coverage, LMIC health systems will need more resources; they will need to be redesigned around the core elements of high-quality primary care. The Starfield ‘4C’ functions of effective primary care—first-contact access, continuity, care coordination and comprehensiveness—offer meaningful targets for policy and planning of primary care in LMICs.^5^ Unfortunately, health systems have not historically been designed or resourced to reliably provide these core functions of primary care.

While access, coordination and comprehensiveness have garnered some policy focus, continuity of care has received relatively little attention within LMICs. Continuity refers to coherent, linked care, between patients, families, communities and providers, across lifetimes. Continuity consists of understanding individuals’ contexts, with longitudinal clinical information, and using this knowledge to build trusting relationships over time.^6^ In higher-income settings, improved continuity has been associated with greater patient satisfaction, improved medication adherence, lower hospitalisation rates and lower mortality.^7^ However, in LMIC literature, there are scant systematic efforts to measure or improve continuity.^7^ Many LMIC health systems provide acute, episodic care, delivered by different providers at different facilities, on a condition-by-condition basis. Such care delivery is increasingly inadequate and outdated as the burden of non-communicable diseases, including mental and behavioural disorders, grows within LMICs.^6^ In this context, a reorientation of care delivery to provide more continuity will be essential to achieve the Sustainable Development Goals and universal health coverage.

## Continuity in primary care: a neglected component of the development agenda

Haggerty and colleagues describe three types of continuity: relational, informational and managerial (table 1).^6^ These three domains of continuity are key components of high-quality primary care, and LMIC health systems must make investments and progress in each in order to achieve high-quality universal health coverage.

**Table 1:** The three types of continuity and examples of each*

	Definition	Intervention	Examples
Relational	An ongoing therapeutic relationship between a patient (and often their family) with one or more providers	Empanelment and multidisciplinary team-based care	Costa Rica’s *Equipos Básicos de Atención Integral en Salud* care team structure^13^
Informational	The use of information on past events and personal circumstances to make current and future care appropriate for each patient and family	Electronic data systems that are interoperable with unique patient identifiers across settings	Public-sector electronic health record system in Nepal^9^
Managerial	A consistent and coherent approach to the management of a patient’s health conditions, that is responsive to changing needs over time	Management standards of care and multidisciplinary team-based care	Patient-centred medical home models in tribal populations in Alaska^14^

*Adapted from Haggerty, *et al* (2003).^6^

### Relational continuity

Of the three types of continuity, relational continuity is most prominently experienced by patients and communities. Relational continuity refers to sustained, healing relationships between patients and providers that cultivate trust and engagement. These relationships are foundational to the improvements in health and well-being that primary care can provide.^6^

In many historically marginalised and impoverished communities within LMICs, the formal health system is often unfamiliar and distrusted. Strong relational continuity can engage these communities, building trust and involving them in improving their health over time.^6^ Recent data from Ghana show improved patient-reported responsiveness outcomes with greater continuity, helping to address negative perceptions of transactional, episodic and fragmented care delivery.^8^ By improving provider consistency, LMICs can build relational continuity that promotes better overall care.

However, attempts to improve continuity exist in tension with access. Increasing continuity may decrease access, and vice versa, leading to a difficult ‘either/or’ dynamic of access versus continuity. For example, to increase continuity for a particular group of patients, their providers may need to limit the overall number of patients they are responsible for, thus potentially decreasing access for some, while improving continuity for others. Understanding these tensions, and aiming for improved—but not perfect—continuity is strategically advisable.

### Informational continuity

Patients experience continuity as a seamless integration of their information by their known providers over time,^6^ promoting a sense of security and trust within these relationships. Without clearly documented and easily accessible information about medical history and demographics—including information about social determinants of health—health systems and providers cannot ensure safe, high-quality services over time.

In many LMICs, where civil registration, vital statistics and health record systems are weak or entirely non-existent, this type of informational continuity is difficult to achieve.^9^ While some LMICs do have robust health management information systems, these serve primarily facility or population-level purposes and do not provide patient-level records, which are commonly relegated to ‘cards’ or ‘booklets’ with scant, encounter-based information that is insufficient to provide informational continuity. Alternatively, these health record systems may exist for certain vertical programmes such as HIV or tuberculosis, but are not well integrated between programmes. Electronic health records are nascent in some places, but rarely scaled across the entire population. Where present, they are often disconnected such that patients have multiple records across care settings, leading to further fragmentation.^9^

Looking forward, significant attention and investments are required to strengthen the infrastructure for patient-level information systems, including centralised, interoperable electronic health records. While these systems by themselves do not guarantee strong informational continuity—and indeed, many high-income settings still struggle to optimise use of electronic health records^10^—they can help to connect individuals’ records across settings as patients are referred or migrate between locations. Empowering both patients and providers, stronger data architecture, whether paper-based or digital, can make informational continuity a feasible reality in settings where, currently, each care episode is distinct and disconnected from past and future.

### Managerial continuity

Managerial continuity is the consistent, coherent management of patients’ health conditions, responsive to changing needs over lifetimes, and across different levels of care.^6^ This requires integrating experiences of care in ways that make sense for patients and families, thus enabling adherence to care plans. Many LMIC health systems, however, already grappling with insufficient workforces and fragmented referral systems, struggle to provide managerial continuity. Accordingly, both additional resources and system redesign with an aim towards integration are required.

Mid-level providers^11^ and community health workers,^12^ operating in multidisciplinary teams,^5^ are practical options to improve access and continuity. Multidisciplinary teams bolster facility-based workforces, and when properly supervised and trained, enable more standardised, integrated care.^11 12^ Examples from Costa Rica^13^ and indigenous communities in Alaska^14^ demonstrate that these multidisciplinary teams can serve poor, rural populations, providing high-quality community-based services, including regular follow-up, referral tracking, medication adherence counselling, risk modification and early warning to clinical worsening.^12^ This type of team-based care enables improved managerial continuity, and is particularly important for patients with multiple chronic conditions, for whom many disconnected episodes of care can lead to complex treatment plans that are difficult to incorporate into their lives.^5 15^

## Pragmatic strategies to improve continuity in primary care

For many LMIC health systems, improving continuity will be greatly challenging, requiring long-term, iterative improvement initiatives aimed at both increasing resources and redesigning care delivery. Nonetheless, there are practical, feasible strategies to improve continuity in these settings, with promising examples already in place in many settings globally.

### Empanelment

Empanelment (also known as ‘rostering’) is the active and ongoing assignment of an individual or family to a primary care provider or team.^5 16^ Empanelment establishes a regular point of care for patients, and holds providers accountable for actively tracking and managing the health of a specific group of individuals. Empanelment also provides a population denominator to enable data tracking, interpretation and iterative improvement of care plans.^16 17^ When done well, empanelment enables both managerial and relational continuity and, when combined with strong data systems, informational continuity as well. For these reasons, empanelment has been heralded as a priority for LMIC health systems by global partnerships such as the Primary Healthcare Performance Initiative (www.improvingphc.org) and the Joint Learning Network (www.jointlearningnetwork.org).^16 17^

In Costa Rica, empanelment systems have been in place for decades, even in the absence of digital data architecture, using community health systems and multidisciplinary teams to provide strong relational, informational and managerial continuity.^13^ Nonetheless, to date, for most LMICs, empanelment is either very weak or completely non-existent.^5^ To achieve the goals of patient-centred universal health coverage, this should be a priority for development partners and governments in future agenda-setting.

### Integrated community-based follow-up

In many LMIC health systems, while empanelment may be a long-term goal, it may not be feasible in the immediate short term. In such settings, programmes aimed at improving retention in care and community-based follow-up after hospitalisation or outpatient visits can offer meaningful improvements in continuity.

Historically, many vertical, disease-specific programmes for HIV/AIDS^18^ and tuberculosis^19^ delivered impressive results in community-based follow-up of patients, improving retention in care over time. More recently, initiatives to ‘horizontalize’ these programmes across a wider spectrum of conditions and patient groups have emerged as well. Data from Uganda^20^ show the feasibility of community outreach workers improving retention in care across a broad range of conditions, including non-communicable diseases, and decreasing overall loss-to-follow-up (LTFU) rates. A public-private partnership in rural Nepal has similarly shown strong continuity over time in a cohort of patients with diabetes, hypertension and chronic obstructive pulmonary disease, using an integrated system of community-based and facility-based care delivery.^21^ These programmes, while not yet at the national level, nor as comprehensive as full empanelment systems, demonstrate feasible, practical short-term options for LMICs to improve continuity in a stepwise manner over time.

## You cannot improve what you do not see

Given that many LMICs currently have minimal primary care continuity, developing better metrics, and then acting on resulting data to improve continuity, is an urgent priority for achieving high-quality universal health coverage. The most commonly used LMIC continuity measure has been LTFU,^20 22^ a metric of success for vertical programmes such as HIV/AIDS and tuberculosis. Policy makers and donors have staked significant resources on achieving low LTFU rates, demonstrating multisectoral alignment for this type of continuity, though the metric has rarely been applied to primary care services.

LTFU rates are, however, a rather crude metric, providing only limited insights into informational and managerial continuity, and little data describing relational continuity. In higher-income settings, more holistic metrics have been developed, attempting to describe all three continuity domains (table 2). While still imperfect, expanded use of these metrics can advance a health system’s understanding of its continuity. To be applicable in LMIC settings, these measurements will need adaptations, including a recognition of the role that multidisciplinary teams play in LMICs, and a concomitant shift away from physician-specific care that is more common in much of Europe and USA.^5 7^ Furthermore, to advance this work for the future, there is a critical need for a dedicated research agenda to better understand both the measurement of, and improvement strategies for, continuity within LMIC health systems.

**Table 2:** Measures of patient continuity*

Measure	Calculation
Provider-sided continuity	% of total visits for a provider with patients on the provider’s panel
Patient-sided continuity	% of total primary care visits for a patient in which the patient sees their empaneled provider
Usual provider continuity	% of all provider visits seen with “usual provider”
Known provider continuity	% of time a patient saw a provider that they had seen previously in the prior year
Continuity of care index	(n^2^ – N) / (N*(N −1)) n = # of visits to a specific provider; N=total # of visits
Sequential continuity	% of time a patient saw the same provider from their previous visit
Modified, Modified Continuity Index (MMCI)	(1 − (P/(V+0.1)))/(1 − (1/(V+0.1)) P=total # of providers; V=total # of visits

*Adapted from Paul, *et al* (2018).^23^

## Conclusion

As the global health community strives towards universal health coverage and the Sustainable Development Goals by 2030, there must be a focus on the key competencies of high-quality, patient-centred primary care delivery. The three domains of continuity deserve more attention, measurement and improvement efforts. Stronger continuity is feasible for LMIC health systems in the short-term and long-term through comprehensive empanelment systems and initiatives such as community-based follow-up programmes to improve retention in care. Continuity enables safer, more effective and more patient-centred care, which are core components of both high-quality healthcare and the universal health coverage agenda. Without improvements in continuity, quality universal health coverage will be impossible. As such, continuity is no longer a goal for high-income countries only; it must become a tracked priority of all health systems to meet their stated ambitions, in order to achieve equitable, high-quality health for all.

## References

[1] World Health Organization Tracking universal health coverage: first global monitoring report. World Health Organization, 2015.

[2] Kruk ME, Gage AD, Arsenault C, et al High-quality health systems in the sustainable development goals era: time for a revolution. Lancet Glob Health 2018;6:e1196–252. 10.1016/S2214-109X(18)30386-3 30196093 PMC7734391

[3] World Health Organization Declaration of Astana, 2018 Available: https://www.who.int/docs/default-source/primary-health/declaration/gcphc-declaration.pdf [Accessed 26 Nov 2018].

[4] United Nations Sustainable Development Goals : Sustainable Development Knowledge Platform. Sustainable Development Goals, 2019 Available: https://sustainabledevelopment.un.org/?menu=1300 [Accessed 4 Apr 2019].

[5] Bitton A, Veillard JH, Basu L, et al The 5S-5M-5C schematic: transforming primary care inputs to outcomes in low-income and middle-income countries. BMJ Glob Health 2018;3(Suppl 3):e001020 10.1136/bmjgh-2018-001020 PMC6169658 30305941

[6] Haggerty JL et al Continuity of care: a multidisciplinary review. BMJ 2003;327:1219–21. 10.1136/bmj.327.7425.1219 14630762 PMC274066

[7] Pereira Gray DJ, Sidaway-Lee K, White E, et al Continuity of care with doctors—a matter of life and death? A systematic review of continuity of care and mortality. BMJ Open 2018;8:e021161 10.1136/bmjopen-2017-021161 PMC6042583 29959146

[8] Ratcliffe HL Patient experience of primary health Care quality and responsiveness in Ghana: results from a nationally-representative survey. Fifth Global Symposium on Health Systems Research, Liverpool, United Kingdom, 2018.

[9] Raut A, Yarbrough C, Singh V, et al Design and implementation of an affordable, public sector electronic medical record in rural Nepal. Jhi 2017;24 10.14236/jhi.v24i2.862 PMC5874496 28749321

[10] Kaiser Health News Death by a thousand clicks: where electronic health records went wrong. Kaiser health news, 2019.

[11] World Health Organization Mid-level health workers for delivery of essential health services. World Health Organization, 2013.

[12] Scott K, Beckham SW, Gross M, et al What do we know about community-based health worker programs? A systematic review of existing reviews on community health workers. Hum Resour Health 2018;16 10.1186/s12960-018-0304-x PMC6097220 30115074

[13] Pesec M, Ratcliffe HL, Karlage A, et al Primary health care that works: the Costa Rican experience. Health Affairs 2017;36:531–8. 10.1377/hlthaff.2016.1319 28264956

[14] Johnston JM, Smith JJ, Hiratsuka VY, et al Tribal implementation of a patient-centred medical home model in Alaska accompanied by decreased hospital use. Int J Circumpolar Health 2013;72 10.3402/ijch.v72i0.20960 PMC3753131 23984283

[15] Hajat C, Kishore SP The case for a global focus on multiple chronic conditions. BMJ Glob Health 2018;3:e000874 10.1136/bmjgh-2018-000874 PMC6035500 29989034

[16] Primary Healthcare Performance Initiative Empanelment | PHCPI. Available: https://improvingphc.org/empanelment [Accessed 26 Nov 2018].

[17] Joint Learning Network Learning from Empanelment in Ghana, 2018 Available: http://www.jointlearningnetwork.org/news/learning-from-empanelment-in-ghana [Accessed 26 Nov 2018].

[18] Nachega JB, Adetokunboh O, Uthman OA, et al Community-based interventions to improve and sustain antiretroviral therapy adherence, retention in HIV care and clinical outcomes in low- and middle-income countries for achieving the UNAIDS 90-90-90 targets. Curr HIV/AIDS Rep 2016;13:241–55. 10.1007/s11904-016-0325-9 27475643 PMC5357578

[19] Law S, Daftary A, O'Donnell M, et al Interventions to improve retention-in-care and treatment adherence among patients with drug-resistant tuberculosis: a systematic review. Eur Respir J 2019;53 10.1183/13993003.01030-2018 30309972

[20] Alizadeh F, Mfitumuhoza G, Stephens J, et al Identifying and Reengaging Patients Lost to Follow-Up in Rural Africa: The “Horizontal” Hospital-Based Approach in Uganda. Glob Health Sci Pract 2019;7:103–15. 10.9745/GHSP-D-18-00394 30926739 PMC6538125

[21] Kumar A, Schwarz D, Acharya B, et al Designing and implementing an integrated non-communicable disease primary care intervention in rural Nepal. BMJ Global Health. 2019;4 10.1136/bmjgh-2018-001343 PMC6509610 31139453

[22] Joshi R, Alim M, Kengne AP, et al Task shifting for non-communicable disease management in low and middle income countries – a systematic review. PLoS ONE 2014;9:e103754 10.1371/journal.pone.0103754 25121789 PMC4133198

[23] Paul K, Ford P, Morris C Continuity of care: how should we measure it?, 2018 Available: https://www.aafp.org/dam/AAFP/documents/events/rps_pdw/handouts/res18-69-continuity-of-care.pdf [Accessed 30 Dec 2018].

